# A dominant, pan-DR binding epitope of Der p 1 in house dust mite allergy induces tolerance in HLA-DR4 transgenic mice

**DOI:** 10.3389/fimmu.2025.1569283

**Published:** 2025-04-11

**Authors:** Heather B. Streeter, Lora G. Lucas, Robert M. West, Mamidipudi T. Krishna, David C. Wraith

**Affiliations:** ^1^ Institute of Immunology and Immunotherapy, University of Birmingham, Birmingham, United Kingdom; ^2^ Leeds Institute of Health Sciences, University of Leeds, Leeds, United Kingdom; ^3^ Department of Allergy and Immunology, University Hospitals Birmingham National Health Service (NHS) Foundation Trust, Birmingham, United Kingdom

**Keywords:** allergy, Der p 1, epitope, immunotherapy, tolerance

## Abstract

**Background:**

Peptides were designed to induce immune tolerance to the major antigen associated with house dust mite (HDM) allergy, Der p 1. HDM is commonly associated with allergic responses in allergic rhinitis and asthma, with Der p 1 specific T-cells implicated in ongoing disease. Tolerogenic peptide immunotherapy can induce tolerance in pathogenic T-cells, bypass mast cell activation and hence reduce the risk of anaphylaxis. A pan-DR binding epitope of Der p 1, covering the broad population, was tested for efficacy in HLA-DR transgenic mice.

**Methods:**

Potential pan-HLA-DR binding tolerogenic T-cell epitopes from Der p 1 were predicted *in silico* and manufactured (synthetic peptides A-E). Participants included HDM sensitised (allergic rhinitis/asthma, n=25), non-HDM sensitised (atopic controls sensitised to ≥1 other aero-allergens, n=10) and non-atopic healthy controls, n=10). Peripheral blood mononuclear cells (PBMC) were collected and screened for immune responses to Der p 1 or test peptides A-E. Mapping of minimal T-cell epitopes, apitope (antigen-processing independent epitope) validation and tolerance induction were tested in HLA-DR transgenic mice.

**Results:**

HDM-sensitised subjects have an elevated response to pan-DR binding peptide D 30mer. Peptide analogue D121B, containing the minimal epitope and optimised for solubility, was verified as a tolerogenic apitope and induced tolerance against Der p 1 antigens in HLA-DR4 transgenic mice *in vivo*.

**Conclusion:**

A tolerogenic peptide, apitope D121B, reduces T-cell immune responses to Der p 1 and is a promising candidate for further development as an immunotherapy for HDM-associated allergic rhinitis and asthma.

## Highlights

- T-cell stimulation was used to identify a dominant epitope from DerP1 antigen in allergic asthma.
- Mapping of minimal epitope in HLA-DR transgenic mice enabled design of a potentially tolerogenic epitope.
- Tolerogenic epitope administration suppressed the immune response to Der p 1 in HLA-DR transgenic mice.

## Introduction

House dust mite (HDM) is the most common indoor aero-allergen associated with allergic rhinitis (AR) and asthma, affecting 1-2% of the global population (1). It is linked to industrialisation, is rising rapidly (2) with a significant economic impact. Sensitisation to HDM strain Dermatophagoides *pteronyssinus* is high in the West (3, 4) and found to be a significant risk factor for asthma in children sensitised <5 years of age (5, 6).

Allergen-Specific Immunotherapy (AIT) has long been used to desensitise patients suffering from allergies (7, 8), including HDM (9, 10). In AIT, increasing doses of allergen extract, administered over time via subcutaneous (SCIT) (11, 12) or sublingual (SLIT) (13–15) routes, modify both innate and adaptive immune responses and can lead to induction of allergen tolerance. Both SCIT and SLIT reduces the progression of AR (16) and may also prevent asthma onset in grass pollen (17) and HDM allergic subjects (18), with improved outcomes in children and in mono-sensitised individuals (19). For sustained immune protection, at least three years of AIT is required (19–21).

Currently, AIT is the only disease-modifying treatment for allergy patients and is now embedded in the treatment of HDM allergy (22). However, all routes of AIT administration (SCIT, SLIT (23), intralymphatic (24)) are associated with local and systemic allergic reactions (25, 26), with an increased risk of anaphylaxis for SCIT. Allergic reactions are directly linked to the administration of whole allergen (or allergen extracts) and their recognition by allergen-specific IgE bound to mast cells. The risk of adverse events together with long courses of treatment impacts on both patient uptake and compliance of AIT (27).

Drawbacks of AIT using whole/extracts of allergen can be averted by substituting with peptide fragments of the allergen. Presentation of dominant T-cell epitope peptides to allergen-specific CD4 T-cells circumvent IgE cross-linking on basophils (28), reducing the chance of an immediate (type-1) hypersensitivity reaction and avoids the risk of anaphylaxis. Peptide AIT with cat dander, birch and grass pollen allergens showed fewer side effects and a good safety profile (29–33). Therapeutic benefits included a rapid reduction of sIgE: sIgG4 ratio (grass pollen (31)), long-term benefits at 2 years (cat dander, Fel d 1 (29)) whilst allergen-specific T-cells responses (32, 34–36) skewed from pathogenic Th2 to more protective tolerogenic responses dominated by regulatory T-cells (Tr1 (34) or Foxp3+ Treg cells (32)).

Previously, we have shown that short peptide epitopes have tolerogenic properties when designed to mimic the naturally processed antigen acting as antigen processing independent epitopes (apitopes) and to be highly soluble (37, 38). Pan-DR epitopes display promiscuity, binding to a wide range of HLA-DR molecules. Thus, selection of one pan-DR antigen-specific peptide can target patients with a wide range of HLA haplotypes. Additional T-cell epitope peptide(s) from the same or other antigens within the disease-inducing allergen can also be identified to create a universal peptide cocktail for treatment. This approach has been successfully employed in peptide immunotherapeutic clinical trials in patients with multiple sclerosis (39, 40) and Graves’ disease (41, 42).

This study focussed on finding pan-DR tolerogenic peptides within Der p 1, a major antigen of HDM. Following an *in silico* search, Der p 1 dominant T-cell epitope peptides were identified in HDM sensitised patients with allergic rhinitis and/or asthma with different HLA haplotypes. Analogues of these peptides were designed and validated as apitopes. One apitope was found to induce tolerance against Der p 1 antigen in HLA-DR4 transgenic mice *in vivo* and is a suitable candidate for testing in AIT for HDM.

## Methods

### Study subjects

Consenting subjects (age ≥18<80 years) were recruited from University Hospital Birmingham NHS FT (details, S1, **Supplementary Table S1**). Selection was based on clinical history, lack of immunomodulatory therapies or corticosteroid treatment (preceding ≤6 weeks) and skin prick test (SPT) to a standard aero-allergen panel including D *pteronyssinus* (HDM). A HDM positive SPT was confirmed by HDM-specific (s)IgE in serum in addition to total serum IgE and Der p 1-sIgE (Immunocap250, S1). Subjects were stratified into 3 groups: HDM-sensitised (HDM SPT and HDM-sIgE positive with allergic rhinitis and/or asthma, n=25), atopic controls (sensitised to one or more aero-allergens but not to HDM, n=10) and healthy non-atopic controls (negative SPTs to HDM and all aero-allergens, n=10).

Peripheral blood mononuclear cells (PBMC) from each subject were isolated and cryopreserved, as previously described (43). The study was approved by North West - Greater Manchester Central Research Ethics Committee (REC 18/NW0726, Protocol number RG_18-207).

### Antigens

Binding predictions of peptides from the Der p 1 sequence to HLA-DR were conducted *in silico* using ProPred and NetMHCII-2.3 programmes (44, 45). Peptides with the highest pan-DR affinity and their related analogues were synthesised by Fmoc chemistry and where required, solubility was optimised by substitution or addition of N-terminal and C-terminal amino acid (aa) tags (**Supplementary Table S2A**). Der p 1 protein was purchased from Citeq Biologics BV.

### Mice

HLA-DR4 transgenic mice, expressing human HLA-DRA*0101, HLA-DRB1*0401 and murine CD3-human CD4 (46) were bred and housed under specific pathogen free conditions in the Biomedical Services Unit, University of Birmingham, in accordance with the local ethical review panel and UK Home Office regulations. Mice (8-16 weeks) were age and sex matched for all experiments.

### Human PBMC assay

Thawed PBMC were re-suspended and cultured with Der p 1 antigen, peptide (A, B, C, D, E or analogues of B or D), *Mycobacterium tuberculosis* purified protein derivative (PPD, positive control) or no antigen (negative control). T-cell proliferative responses were evaluated on days 4-8 by measuring incorporation of 3[H]-thymidine (43), expressed as Stimulation Index (SI) (positive response cpm >1000 and Stimulation Index (SI) >3 (SI: cpm with antigen/cpm no antigen) (43), S2.D.1).

### Mouse immunisation and *in vitro* recall assay

Mice were immunised with antigen Der p 1 (test) or PBS (control) in Complete Freund’s adjuvant (CFA), injected subcutaneously (s.c) in the lower dorsal region (S2.C). After ten days, mice were sacrificed and splenocyte T-cell responses were evaluated by the secretion of interferon gamma (IFN-γ) into culture medium after 72h (ELISA, S2.D.2).

### Peptide apitope validation

Presentation of antigen (Der p 1 or peptides) by fixed (test) or unfixed (control) HLA-DR4 mouse splenocytes was determined following activation of a Der p 1-specific T-cell hybridoma (S2.B), and subsequent IL-2 secretion (ELISA, S2.D.3) *in vitro*. Presentation by fixed APC indicates that the peptide does not require further processing.

Direct binding of peptide D121B to MHC Class II on mouse HLA-DR4 splenic dendritic cells (DC) *in vivo* was determined 2h post injection (s.c.): isolated CD11c+ DC were isolated and incubated with a Der p 1-specific T-cell hybridoma and activation assessed by IL-2 secretion (ELISA, S2.D.4).

### Induction of tolerance in mice

HLA-DR4 transgenic mice received escalating doses (0.1ug, 1ug, 10ug, 3 x 100ug) of peptide D121B (test) or PBS (control), by s.c. injection (scruff of the neck) every 3^rd^ or 4^th^ day with the final dose on day 28. Three days later test and control mice were immunised with 100ug of peptide D in CFA (s.c. lower dorsal area, S2.C). After 10 days, splenocytes were harvested and restimulated *in vitro* by culturing with various antigens for 3 days (S2.D.2). T-cell stimulation was assessed by IFN-γ secretion (ELISA).

### Statistical analysis

PBMC responses to allergens in HDM sensitised and HDM non-sensitised (combined non-HDM sensitised atopic and healthy non-atopic controls) were compared in a cross-sectional study design devised in R (47), only main effects were considered in this exploratory work (details in S2.E). Significance of t-tests (Satterthwaite) was set at *p<0.05, **p<0.01, ***p<0.001.

For mouse tolerance experiments, data was analysed with an unpaired Student’s t-test with Welch’s correction using GraphPad Prism 9.0. Significance levels were set at *p<0.05, **p<0.005, ***p<0.0005.

## Results

### Selection of tolerogenic T-cell epitopes

**Figure 1:**
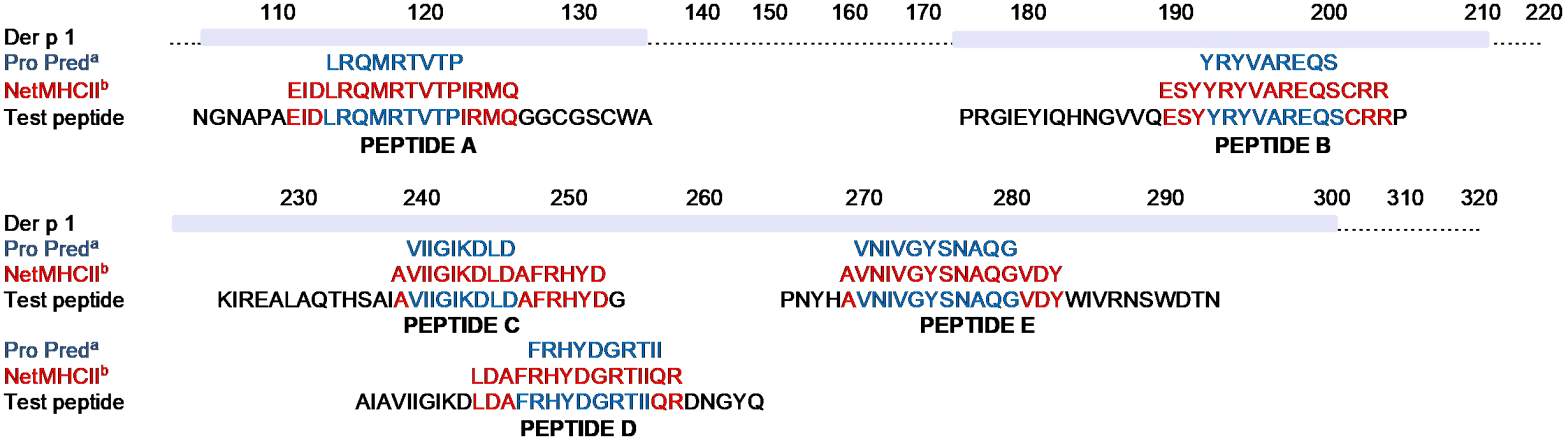
*In silico* prediction of Der p 1 T-cell epitopes. Pan-DR binding predictions of T-cell epitopes within the Der p 1 aa sequence to HLA-DR was determined by either ProPred (a, blue font) or NetMHCII-2.3 (b, red font) programmes. Core sequences of peptides with the highest pan-DR affinity, identified by both prediction programmes, were selected from each of 5 different regions of Der p 1. Addition of flanking amino acids based on the native sequence of the Der p 1 protein (black font) were added to the core sequence to generate 5 peptides of 29-30 amino acid length (Peptide A-E) and synthesised by F-moc chemistry.

T-cell epitopes were predicted according to MHC class II binding affinity. The highest affinity pan-HLA-DR peptides predicted by both ProPred and NetMHCII programmes were selected based on the 9-mer or 15-mer core sequences (**Figure 1**). Test peptides were designed to span the predicted core epitope by adding flanking amino acids from the Der p 1 protein. Five peptides of 29-30 amino acid (aa) length were identified as potential T-cell epitopes (A to E, **Figure 1**).

### Serum IgE analysis of subjects

**Figure 2:**
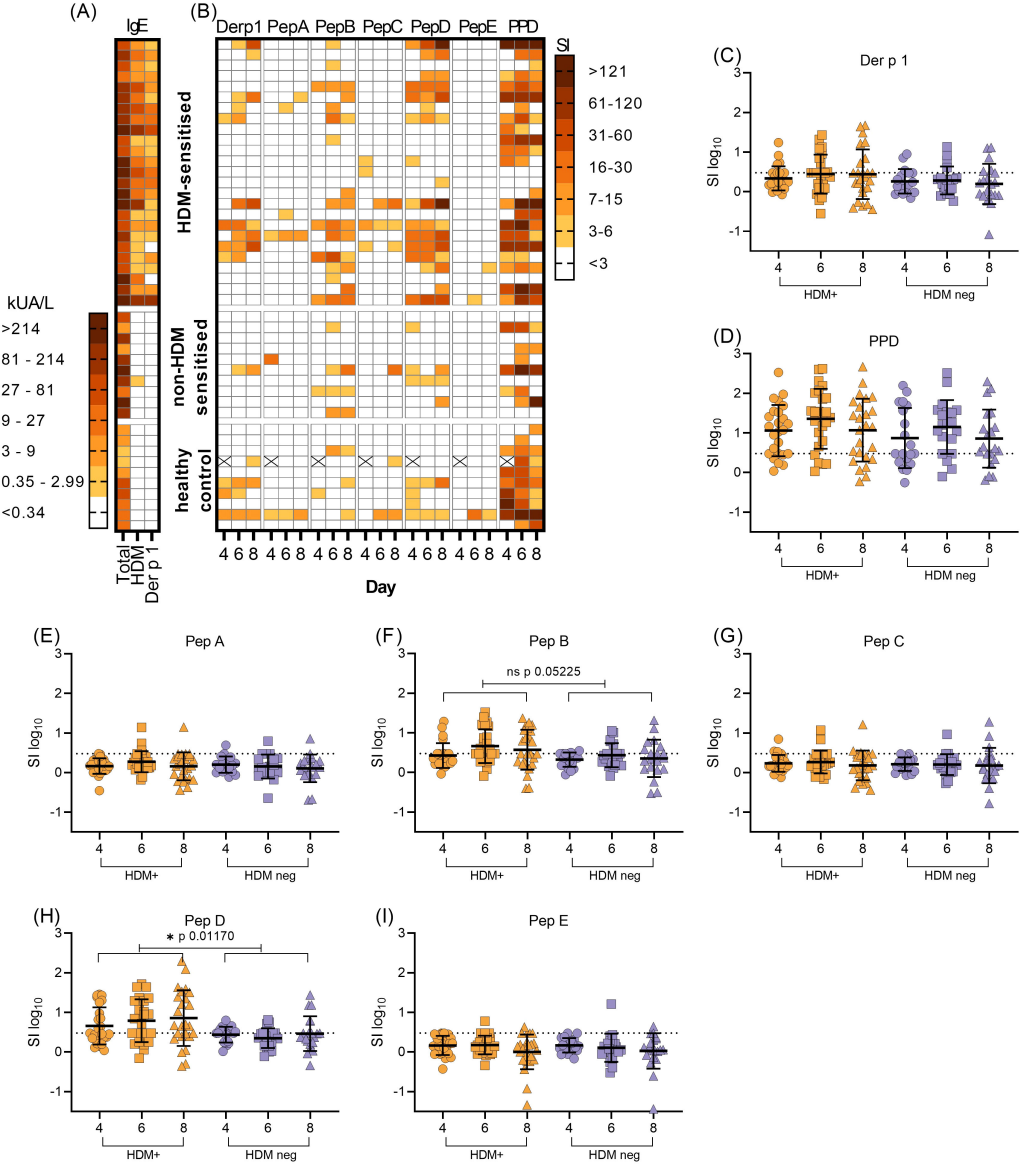
Serological and T-cell responses to HDM, Der p 1 and Der p 1-derived peptide epitopes. **(A)**, the level of total serum IgE was compared with HDM and Der p 1- sIgE in HDM allergic (n=25), non-HDM allergic (n=10) and healthy control (n=10) subjects (KUA/L). **(B)**, corresponding T-cell proliferative responses to Der p 1, Der p 1-derived synthetic peptides A-E and PPD was measured by the incorporation of [3H]-thymidine in PBMC cultures on day 4, 6 and 8. Proliferation was expressed as stimulation index (SI). Missing values due to low cell yield are denoted by (X). **(C–I)**, expression of T-cell proliferation as transformed stimulation index (SI_log10_, mean+/-SD), in HDM-positive (HDM-sensitised) PBMC (orange symbols) compared with HDM-negative PBMC (combined non-HDM sensitised and healthy control groups, blue symbols) on each day of assay. Values above the dotted horizontal line on each graph denote positive T-cell responses to antigens Der p 1 **(C)**, PPD **(D)** or Der p 1-derived peptide epitopes **(E–I)**. Statistical analysis was as described in Methods and S1, using Satterthwaite’s method for t-tests, with significance at *p<0.05, **p<0.01, ***p<0.001.

Most subjects showed concordance between HDM sensitisation as detected by the SPT and serum sIgE. All subjects within the HDM-allergic group were positive for HDM-sIgE (**Figure 2A**), this group displayed the highest level of both HDM-specific and total serum IgE (**Figure 2A**). Individuals in the non-HDM sensitised atopic positive control group had no (9/10) or very low levels of HDM-specific IgE (0.58 kUA/L) with none in the healthy control group (0/10). Most HDM-allergic subjects had sIgE antibodies specific for Der p 1, a HDM constituent protein. (23/25). In contrast, no Der p 1-sIgE was found in either non-HDM sensitised atopic positive control or healthy non-atopic controls.

### Human T-cell responses to Der p 1 and Der p 1-derived epitopes

Screening of PBMC proliferation in the presence of Der p 1 antigen or *in silico* selected and designed pan-HLA-DR test peptides A to E (**Figure 1**) revealed variation in peak day T-cell responses (day 4, 6 or 8, **Figure 2B**), as previously observed in this kinetic *in vitro* assay (39). T-cell responses to peptides B and D were elevated and more frequent (15/25 and 17/25 respectively) in the HDM-sensitised group compared with the non-HDM-sensitised groups (**Figure 2B**).

Some subjects demonstrated a T-cell response to Der p 1 antigen, in all 3 study groups (**Figure 2B**), despite lacking a Der p 1 sIgE. For the two HDM sensitised individuals with negative Der p 1 sIgE, T-cells were activated with peptides B or D (SI 3-15) and strongly with Der p 1 in one subject (SI>16) (**Figure 2B**). Most individuals demonstrated a clear response to the positive control antigen PPD; non-responders were characterised by either weak T-cell responses to all other test antigens (no response or SI >7) or had not been inoculated with BCG (PPD SI>3 on any assay day) (**Figure 2B**). Non-PPD responders were distributed evenly between HDM sensitised and non-HDM sensitised groups. Initial observations indicated no distinct differences between T-cell responses to Der p 1 or Der p 1-derived test peptides in the non-HDM sensitised or healthy control groups; therefore, these were combined into one group for statistical analysis, described henceforth as the HDM-negative group.

Variation between HDM-positive and the HDM-negative group was significant for Peptide D at the 5% level (*p0.0117, t2.633, **Figure 2H**). Furthermore, there was a trend towards significance between test and control responses to Peptide B (p0.0525, t1.994, **Figure 2F**). Little evidence of differences appeared between groups responding to other antigens (**Figures 2C–E, G, I**), since the reported *t* values were small in absolute value (**Supplementary Table S2**). Preliminary analysis of HLA DR type indicates that indeed, both peptides D and B are pan-DR binding, with no over-representation of any particular allele. The response to peptide D was clear and there was a trend towards a response to peptide B; this warranted further study of both peptides in an HLA-DR transgenic mouse model.

### Minimal T-cell epitope of peptide D

To define T-cell epitopes residing within peptide B or D, a panel of overlapping 15-mer peptides shifting in sequence by 3 aa was used to fine map these regions using splenocytes from HLA-DR4 transgenic mice.

**Figure 3:**
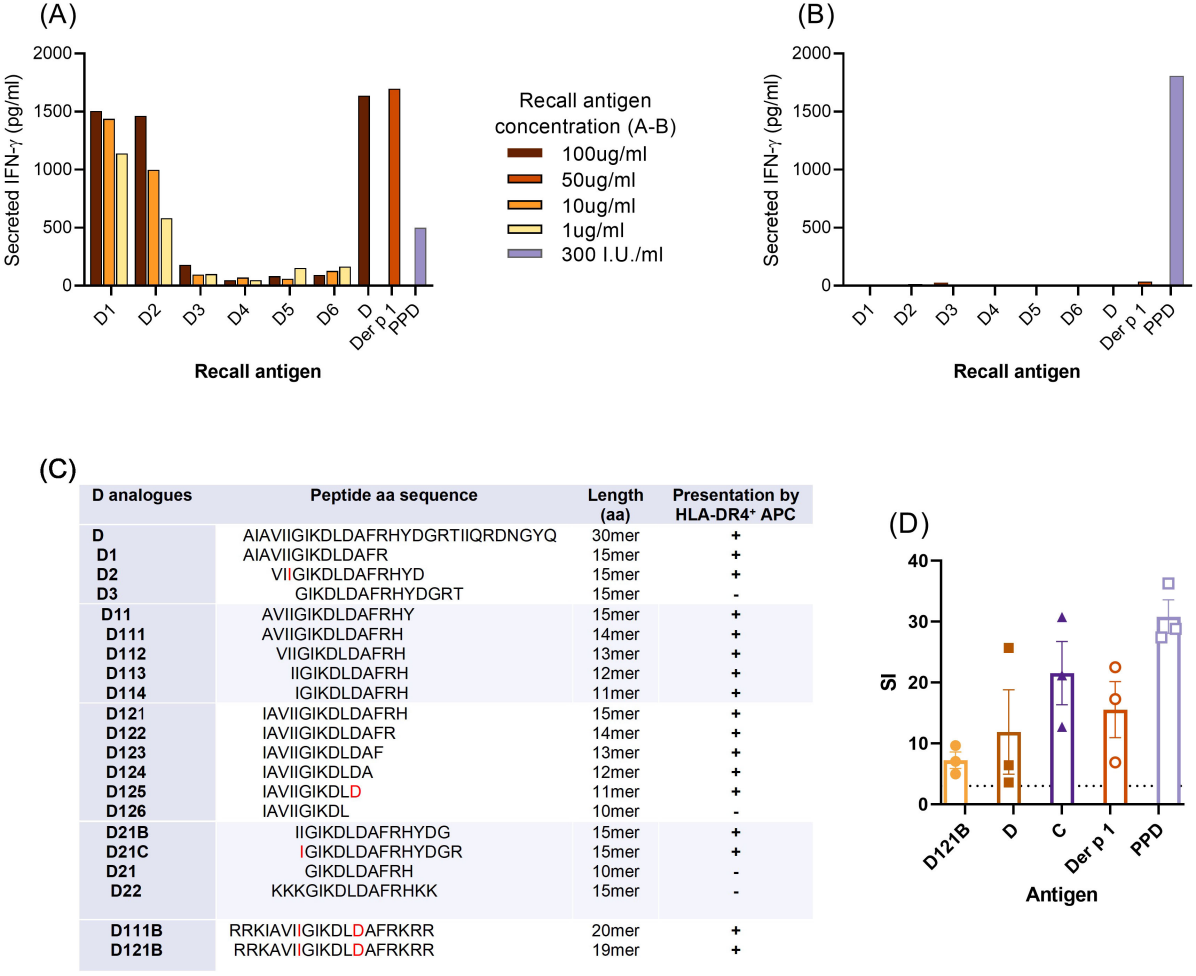
Minimal T-cell epitope of peptide D. **(A–C)**, For fine-mapping of T-cell epitope(s), splenocytes from individual HLA-DR4 mice immunised with Der p 1 **(A)** or PBS in CFA **(B)**, were re-challenged *in vitro* with peptide D, analogues D1-D6 (15mer peptides, overlapping by 3aa), Der p 1 or PPD, for 72h. T-cell stimulation was measured by IFN-γ secretion (pg/ml, ELISA) for each antigen in triplicate wells and results shown are representative of 3 individual experiments. **(C)**, defining the minimal T-cell epitope of peptide D; summary of T-cell IFN-γ secretion (+/-) in response to truncated analogues (overlapping by 1aa) of D1 and D2. Boundaries of the minimal T-cell epitope sequence are in red font. **(D)**, proliferative response of human HLA-DR4 PBMC (HLA DRB1*04:01 DRB1*14:54) to analogue D121B, containing the minimal T-cell epitope. Proliferation was measured by the cell incorporation of [3H]-thymidine in triplicate wells after 7 days of culture with antigens D121B, D, C, Der p 1 (all at 20ug/ml) or PPD (300I.U/ml) and expressed as stimulation index (SI); values above the dotted horizontal line denote a positive T-cell response to test or control antigens.

Splenocytes from mice immunised with Der p 1 responded to Der p 1, peptide D and two of the N-terminal 15-mer peptides from peptide D (D1 and D2) in an *in vitro* recall assay (**Figure 3A**). No recall responses were observed with these antigens following immunisation with CFA alone (**Figure 3B**). This confirms that T-cell responses to peptide 15mers D1 (aa 1-15) and D2 (aa 4-19) were specific when presented to HLA-DR4. Furthermore, it suggests that the minimal T-cell epitope in the N-terminal region resides within aa sequence 4-15 of peptide D.

To identify the minimal T-cell epitope within peptide D, T-cell responses to a set of overlapping (1aa) and truncated peptide analogues of D1 and D2 were evaluated in splenocyte assays. The core sequence was defined as a 7mer, IGIKDLD, with 2 analogues D111B and D121B with their solubility optimised by addition of hydrophilic amino acids (**Figure 3C**). Analogue D121B induced a proliferative response in PBMC from a Der p 1, HLA DR4 (HLA DRB1*04:01 DRB1*14:54) positive individual (**Figure 3D**) indicating that it is a relevant epitope in humans. Furthermore, the PBMC also responded to peptide C, which has an overlapping sequence with D121B (**Figure 1**).

Despite positive responses to peptide B, none of the shorter overlapping 15-mer B peptides could activate splenocytes in the *in vitro* recall assay (**Supplementary Figure S1**). Thus, only analogues D1 and D2 could be assessed for peptide immunotherapy in the HLA-DR4 mouse model.

### Validation of D121B as an apitope in HLA-DR4 transgenic mice

**Figure 4:**
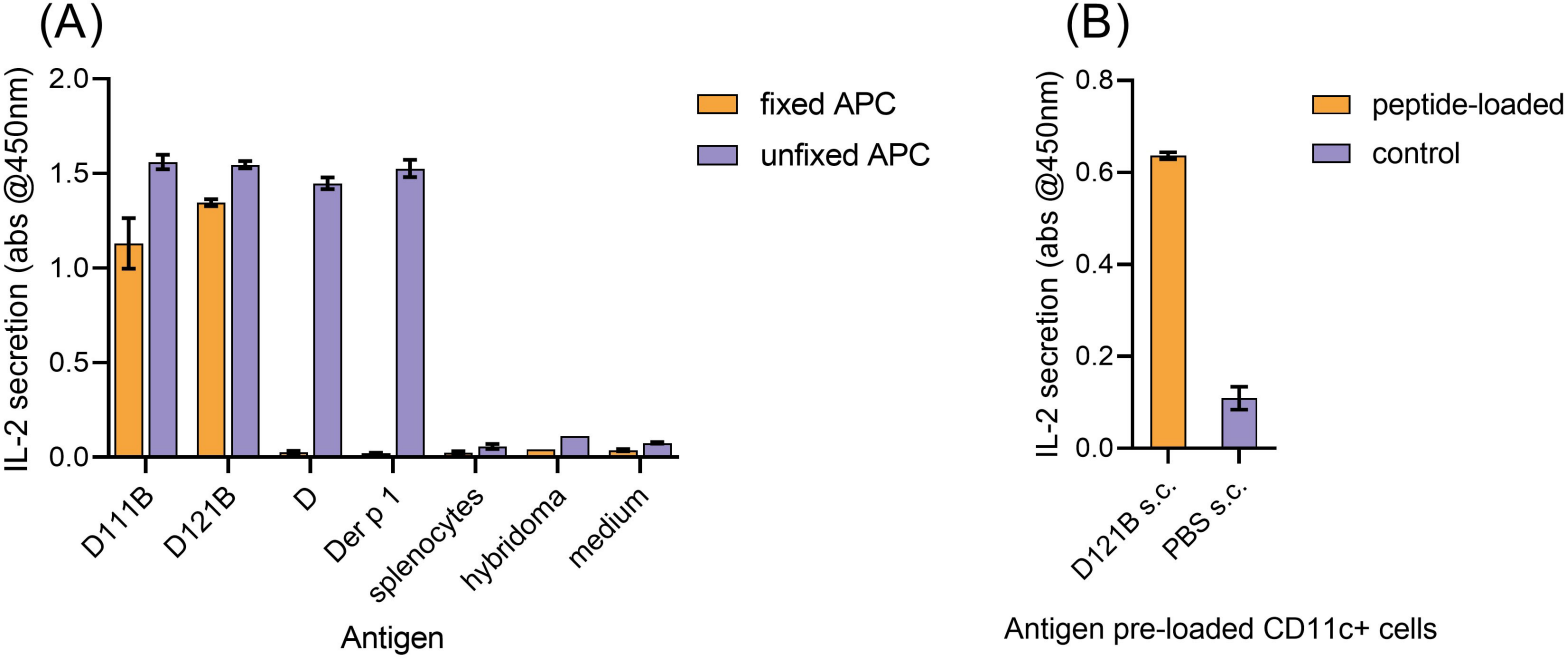
Validation of D peptide analogues as apitopes. **(A)**, *in vitro* presentation of antigen (D111B, D121B, D or Der p 1) by fixed HLA-DR4 mouse splenocytes (direct binding, no processing, orange bar) or unfixed splenocytes (blue bar) following co-culture with Der p 1-specific T-cell hybridoma cells. Activation was evaluated by IL-2 secretion (pg/ml, ELISA) after 24h. Control wells contained splenocytes, hybridoma, or no antigen (medium). **(B)**, direct binding of D121B to MHC Class II on CD11c+ cells *in vivo*. Two hours post-injection (s.c) of HLA-DR4 mice (2 per group) with a single dose of peptide D121B (test, orange bar) or PBS (control, blue bar), splenocyte CD11c+ cells were isolated and co-cultured with Der p 1 specific T-cell hybridoma cells *in vitro*. T-cell hybridoma cells secreted IL-2 (pg/ml) in response to *in vivo* peptide pre-loading of CD11c+. Graphs A and B show mean of triplicate wells ± SEM, representative of 2 experiments.

The potential of peptides D111B and D121B as tolerogens was evaluated by their apitope characteristics. Apitopes do not require processing, thus can be presented effectively to T-cells by fixed APC. Presentation of peptide D, Der p 1, D111B and D121B by live naïve HLA-DR4 mouse splenocytes (APCs) resulted in IL-2 secretion by a Der p 1 hybridoma, but none in control cultures (**Figure 4A**, unfixed APC). However, upon fixation of APC, neither the 30-mer peptide D nor the intact antigen Der p 1 could activate the hybridoma, whereas analogues D111B and D121B both elicited an IL-2 response (**Figure 4A**, fixed APC). This provides evidence that epitopes D111B and D121B bind directly to MHC II without further antigen processing and therefore function as apitopes.

To induce tolerance, previous studies have shown that an apitope must be soluble and bind directly to HLA-DR on steady state CD11c+ DC in lymphoid organs (38). Following a single s.c injection of peptide D121B in an HLA-DR4 mouse, a Der p 1-specific T-cell hybridoma was activated *ex vivo*, showing that peptide D121B had indeed bound to splenocyte CD11c^+^ cells *in vivo* (**Figure 4B**). No response was seen with CD11c^+^ DCs isolated from PBS treated mice (**Figure 4B**); however, cells maintained their ability to present Der p 1 and peptides when antigens were added *ex vivo* (**Supplementary Figure S2**).

Both the *in vitro* fixed APC assay and the *in vivo* CD11c+ binding experiments validate peptide D121B as an apitope, thus a suitable candidate tolerogen.

### Peptide D121B induces tolerance in HLA-DR4 mice

**Figure 5:**
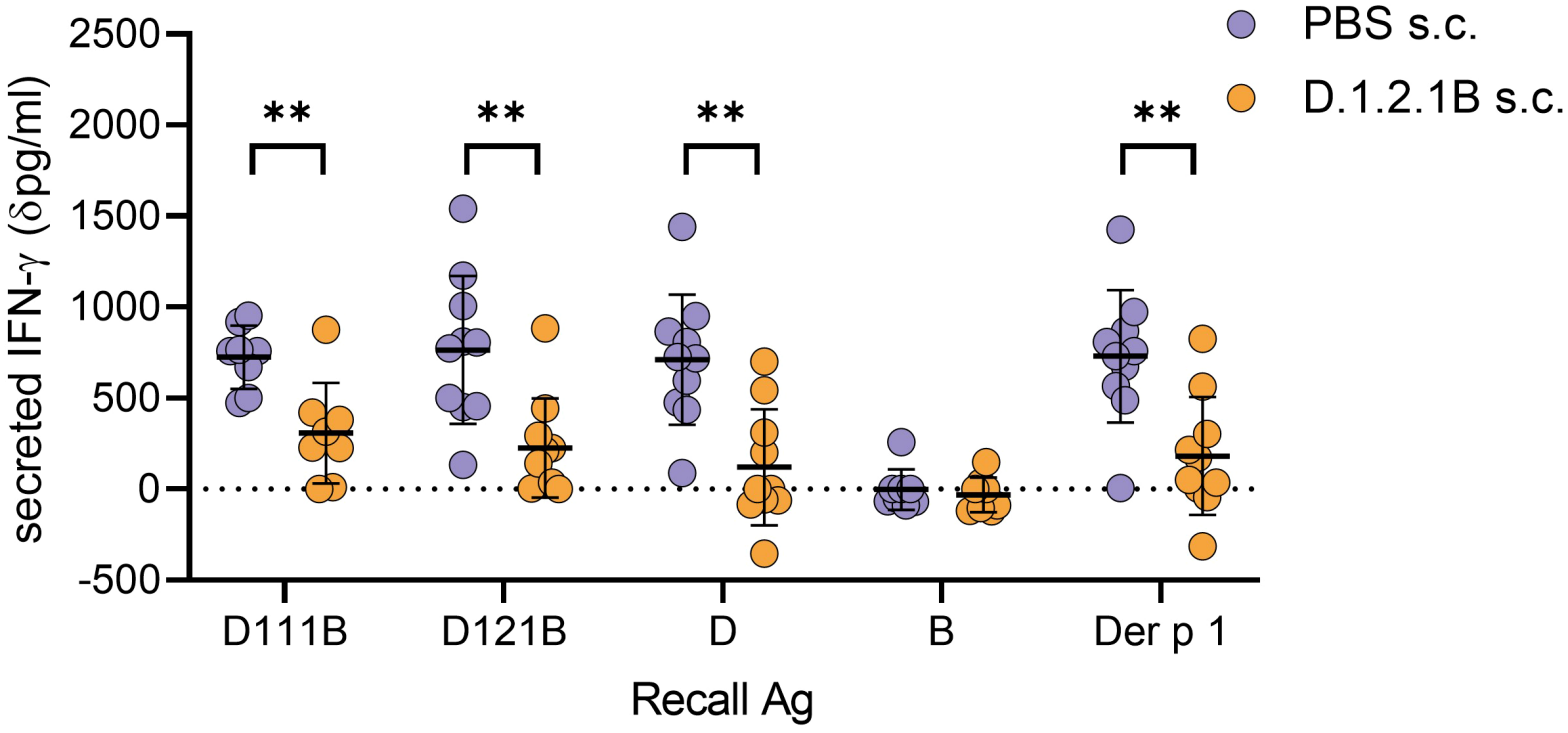
Induction of tolerance with peptide D121B in HLA-DR4 mice. HLA-DR4 mice received escalating s.c. doses of either PBS containing 0.1ug, 1ug, 10ug and 3 x 100 ug D121B peptide (test, orange circles) or PBS alone (control, blue circles) every 3^rd^ or 4^th^ day. Three days after the last dose both test and control mice were immunised s.c. with 100ug of peptide D in CFA. Ten days later, splenocytes were harvested and restimulated *in vitro* for 72h with either D111B, D121B, D, B (1ug/ml), Der p 1 (10ug/ml) or no antigen. Responses were evaluated by IFN-γ in culture supernatant (dpg/ml: IFN-γ pg/ml antigen containing culture – IFN-γ pg/ml culture without antigen, ELISA). Graph shows the mean value ± SD for 10 samples per treatment group collected from three experiments. Data was analysed with an unpaired t-test with Welch’s correction, *p<0.05, **p<0.005, ***p<0.0005, GraphPad Prism.

Administration of repeated doses of a soluble apitope, by dose escalation, induces tolerance associated with T-cell anergy and Treg cell generation (48, 49). Dose escalation with D121B prior to immunisation with peptide D in HLA-DR4 transgenic mice resulted in a suppressed immune response in splenocytes re-stimulated *in vitro* with Der p 1 or peptide D analogues as compared with PBS controls (**Figure 5**). As expected, no response was observed in D121B or PBS treated splenocytes re-stimulated with peptide B. Treatment with the apitope D121B led to reduced IFN-γ secretion to the homologous peptide D121B (p0.0032), overlapping peptide D111B (p0.0036), peptide D (p0.0011) and Der p 1 (p0.0022). This provides evidence that the pan-DR binding apitope D121B should be considered as an appropriate tolerogen to mediate antigen-specific immunotherapy in individuals with HDM-induced allergic rhinitis/asthma.

## Discussion

Our *in silico* analysis, using two distinct peptide binding algorithms, predicted and identified pan-DR T-cell epitopes of Der p 1 (**Figure 1**), some overlapping with previously described epitopes (50, 51). There are multiple elements determining the immunodominance of T-cell epitopes from a given antigen in humans. These include HLA class II binding affinity, the ability of human APC to reveal the epitope during antigen processing and finally the presence of T-cells carrying a TCR complimentary to the conformation created by binding of the peptide epitope to a specific MHC class II molecule. Having identified epitopes by two independent algorithms, we have extended the predicted epitopes to create long (~30mer) peptides to optimise the generation of naturally processed epitopes from the longer peptide. It is important for us to design pan-DR binding epitopes since, unlike in many autoimmune diseases, MHC is less closely associated with allergic asthma.

We compared immune responses to Der p 1 and the five predicted pan-DR binding epitopes (**Figure 2**). This revealed a correlation between recognition of Der p 1 and epitopes B and D with a significant increase in the response to D among subjects sensitised to house dust mite and a trend in response to B. Some individuals responded to peptides B and D without recognition of Der p 1. One explanation would be that the peptide sequences are shared with other Der p antigens; however, there is no homology between peptide D and Der p 2, Der p 5, Der p 7 or Der p 23. Alternatively, it is possible that the APC found in our PBMC cultures are not optimal for processing of epitopes B and D from the intact antigen. The most likely explanation, however, is that the concentration of Der p 1 protein in our cultures was too low to elicit a response in these individuals.

Immunisation of HLA-DR4 transgenic mice with Der p 1 led to a strong immune response to both peptide D and the intact Der p 1 protein itself (**Figure 3**). These mice do not express endogenous mouse class II MHC proteins (46). This proves that peptide D contains a naturally processed epitope from Der p 1 identity of which was further mapped to the N-terminus of the peptide. It was not possible to identify an epitope from peptide B. This is most probably because the epitope created by processing of the whole peptide B cannot be recreated by any of the shorter peptide sequences. We have previously found evidence of such dominant cryptic epitopes in other antigens (52).

The dominant epitope from peptide D in HLA-DR4 transgenic mice mapped to residues IGIKDLD which is predicted to be a pan-DR binding epitope (**Figure 4**). In addition to HLA-DR binding predicted for DRB1*0301, *0404, *0701 and *1401, the NetMHCII pan algorithm shows that D121B is predicted to bind specific HLA-DQ molecules (DQA10102-DQB10501; DQA10102-DQB10604; DQA10103-DQB10501; DQA10103-DQB10603) further broadening its specificity and likely impact on tolerance induction. Importantly, the IGIKDLD sequence is found in both peptides C and D which would explain why there is recognition of both peptides in some individuals (**Figure 2**). The fact that some other HDM-sensitised individuals respond to peptide D and not peptide C requires explanation. First, it could be that this epitope is more readily processed and presented from peptide D rather than peptide C. The fact that those individuals responding to both peptide D and C always respond more strongly to peptide D would be consistent with this explanation (**Figure 2**). Secondly, it may well be that there is a second T-cell epitope within peptide D that is not identified following immunisation of HLA-DR4 mice with this peptide. We have sought an explanation based on the HLA type of those responding to both peptide C and D versus those responding only to peptide D and cannot provide a logical explanation based on HLA type. It is, therefore, more likely that there is a second T-cell epitope in peptide D that fails to be recognised by the mouse T-cell receptor repertoire selected in the thymus of HLA-DR4 transgenic mice (53).

Our previous work defined the rules governing the design of tolerogenic peptide epitopes. The first rule is that the peptide must mimic the naturally processed T-cell epitope to induce tolerance in those T-cells specific for Der p 1 (52). This is confirmed by demonstrating that the peptide functions as an antigen processing independent T-cell epitope (**Figure 4**). Secondly, we have shown that tolerogenic T-cell epitopes selectively bind to unstable MHC II molecules on steady-state dendritic cells (38). These cells are known to induce tolerance primarily because they express low levels of costimulatory molecules (54). We have previously shown that tolerogenic peptides must be highly soluble to reach steady-state dendritic cells in lymphoid organs (38). Peptide D121B was, therefore, designed to function as an apitope, was modified to optimise solubility and hence shown to bind directly to CD11c, steady-state dendritic cells following subcutaneous injection. Finally, proof that D121B functions as a tolerogenic peptide was provided by tolerance experiments in HLA-DR4 transgenic mice. These experiments showed that D121B suppressed the immune response to both peptide D and Der p 1 showing that this apitope was capable of tolerising T-cells specific for the naturally processed epitope. Further work is required to prove that D121B can induce tolerance in HDM-sensitised individuals.

Our approach using *in silico* selection of pan-DR binding epitopes is clearly successful for rapid identification of potentially tolerogenic T-cell epitopes. The design of apitopes from such predicted epitopes has had proven success in autoimmune diseases with distinct immune pathologies (39–42). Here we show that an apitope from Der p 1 can be used to suppress the immune response to the antigen in a relevant HLA-DR transgenic mouse. We propose that our approach can be extended to create cocktails of peptides from known allergens that would provide safe and cost-effective treatments for allergic diseases. Apitopes have a good safety profile in the four clinical trials conducted to date in autoimmune diseases (39, 40, 42). Combined with the markedly reduced risk of IgE binding and anaphylaxis associated with the use of short peptides for immunotherapy, we believe that apitope immunotherapy would improve patient compliance with treatment and help slow the progression of diseases such as allergic rhinitis and asthma.

## Conclusion

A tolerogenic peptide, apitope D121B, reduces T-cell immune responses to Der p 1 and is a promising candidate for further development as an immunotherapy for HDM-associated allergic rhinitis and asthma.

## Data Availability

The original contributions presented in the study are included in the article/**Supplementary Material**, further inquiries can be directed to the corresponding author/s.
