# Supplementary material for: A dominant, pan-DR binding epitope of Der p 1 in house dust mite allergy induces tolerance in HLA-DR4 transgenic mice
Source: Front Immunol. 2025 Apr 11;16:1569283. doi: 10.3389/fimmu.2025.1569283 (PMC12021919; doi:10.3389/fimmu.2025.1569283)
Supplement: Supplementary file 1 [file DataSheet1.docx]

**Supplementary Information**

**S1: Study subjects and inclusion criteria**

Subjects (age ≥18<80 years) were identified from clinical and laboratory databases and allergy/asthma clinics in University Hospital Birmingham (UHB) NHS Foundation Trust, UK. Healthy non-atopic controls were identified *via* local advertisement at UHB. Subjects were excluded from the study if they were on immunomodulatory therapies (eg. omalizumab, anti-TNF etc) or received regular/recent oral corticosteroid treatment (preceding ≤6 weeks), had a significant psychiatric condition, were smokers or unable to give informed consent.

Initial subject selection was based on clinical history and responses to a skin prick test (SPT) to a standard aero allergen panel of D *pteronyssinus*, D *farina*, grass pollen, tree pollen (silver birch), cat, dog and aspergillus (ALK Abello).  An allergen response with a wheal diameter ≥5mm greater than negative control was deemed positive. An HDM SPT response was confirmed by the presence of HDM-specific (s) IgE in serum (, Phadia, ImmunoCap250). In addition, serum levels of Der p 1-sIgE and total IgE were evaluated (ImmunoCap 250). Based on these analyses, subjects were stratified into 3 groups:

- HDM-sensitised (HDM SPT and HDM-sIgE positive with allergic rhinitis and/or asthma, n=25)
- non-HDM sensitised (negative HDM SPT but positive to ≥1 aero-allergen (atopic positive control), n=10)
- healthy non-atopic negative controls (negative SPTs to HDM and all aero-allergens, n=10).

Peripheral blood mononuclear cells (PBMC) were isolated by density gradient centrifugation (Ficoll® paque PLUS) from a citrated peripheral blood sample collected from each subject and cryopreserved in liquid nitrogen, as previously described^42^.

All subjects provided written informed consent. The study was approved by the North West - Greater Manchester Central Research Ethics Committee (REC reference 18/NW0726, Protocol number RG_18-207).

**Table S1: Age, Gender and IgE status of study subjects**

|  | **Group** | | |
| --- | --- | --- | --- |
|  | **HDM-sensitised** | **Non-HDM sensitised**  **(positive control)** | **Healthy non-atopic control**  **(negative control)** |
| **Age** (mean + SD) | 36.4 (+11.76) | 35.5 (+11.75) | 47.0 (+9.23) |
| **Gender** (Male (M)/ Female (F) mean + SD) | M 35.73 (+11.63)  F 37.71 (+12.22) | M 50.50 (+9.20)  F 31.75 (+9.23) | M 43.50 (+17.68)  F 47.88 (+7.77) |
| **Total IgE** Ku(median)  25% percentile  75% percentile | 109  41.50  428.00 | 87.50  11.75  223.00 | 9.00  4.29  23.75 |
| **HDM IgE** (median)  25% percentile  75% percentile | 9.39  3.06  29.35 | 0.05  0.00  0.14 | 0.00  0.00  0.013 |
| **Der p 1 IgE** (median)  25% percentile  75% percentile | 3.11  0.91  9.935 | 0.00  0.00  0.015 | 0.00  0.00  0.00 |

(positive sIgE ≥0.35 kUA/L)

**S2: Methods**

**S2.A: Reconstitution of antigens**

Lyophilized synthetic peptides were reconstituted in dimethyl sulfoxide, stored at -80^o^C, then thawed and diluted in phosphate buffered saline (PBS) as indicated.

Der p1 protein (Citeq Biologics BV, Groningen, Netherlands) was reconstituted in PBS and diluted as indicated.

**S2.B: Generation of Der p 1 T cells hybridomas**

T-cell hybridomas were generated as described(Canaday, 2013). Briefly, splenocytes isolated from HLA-DR4 mice immunized with Der p 1 (50μg/mouse) were re-stimulated *ex vivo* with Der p 1 protein at 50μg/ml in supplemented X-VIVO 15 medium for 5 days. Following fusion with thymoma BW5147 cells (kind gift of Dr W Born, University of Colorado), Der p 1-specific T cell

To test for Der p 1 specificity, expanded hybridoma clones were co-cultured with DR4-expressing Priess cells (HLA DRB1: 04 and HLA DRB4: 101, EACC #8605211) and Der p 1 antigen for 24h; activation of T cell clones was detected by IL-2 secretion in culture supernatant (ELISA, BioLegend).

**S2.C: Mouse immunisation**

Mice were immunised with an emulsion of either Der p 1 (50μg), peptide B (100μg) or peptide D (100μg) in 100ul Complete Freund’s adjuvant (CFA) supplemented with 400ug heat-killed Mycobacterium tuberculosis H37RA, injected subcutaneously (s.c.) at two sites in the lower dorsal region (50μl/site). Control mice were immunised with PBS alone in CFA with H37RA. Ten days after immunisation, mice were sacrificed and spleens collected.

**S2.D: *In vitro* assay conditions**

1. **Human PBMC assay**

**Medium**: Thawed PBMC were cultured in RPMI 1640 medium supplemented with 20mM HEPES, 100U/ml Penicillin, 100μg/ml streptomycin and 10% heat-inactivated autologous plasma^40^, cultured in a 24 well plate at 1.2 X 10^6^ cells/well at 37^o^C/5%CO_2_.

**Antigens**:

- Test - Der p 1, peptide A, B, C, D or E, peptide analogues of B or D at 20μg/ml
- Positive control - *Mycobacterium tuberculosis* purified protein derivative (300 I.U/ml, PPD, Bovigam, Thermo Fisher (positive control)
- Negative control - no antigen (medium alone)

1. **Assay readout**: T-cell proliferation, kinetic assay, monitored in duplicate aliquots of cell suspensions on days 4, 6 and 8 by measuring cell incorporation of 3[H]-thymidine, as previously described(Mazza et al., 2002). Proliferation was considered positive when cell incorporated cpm >1000 and Stimulation Index (SI) >3 (SI: cpm with antigen/ cpm no antigen).
2. **Assay readout to assess response to analogue D121B**: essentially as above, except T-cell proliferation was assessed in triplicate aliquots of cell suspensions from triplicate culture wells/antigen on day 7.
3. ***In vitro* recall assay following mouse immunisation**

**Medium**: Disaggregated splenocytes were seeded in triplicate in X-VIVO 15 supplemented with 100U/ml Penicillin, 100μg/ml streptomycin, and 0.05μM 2-mercaptoethanol

at 0.5 X 10^6^ cells in 96-well flat-bottomed tissue culture plates at 37^o^C/5%CO_2_ for 72h

**Recall Antigens**:

- Test - Der p 1 (10-50ug/ml) or peptides (1-100μg/ml)
- Positive control - *Mycobacterium tuberculosis* purified protein derivative (300 I.U/ml, PPD, Bovigam, Thermo Fisher (positive control)
- Negative control - no antigen (medium alone)

**Assay readout**: T cell responses were evaluated by the secretion of interferon gamma (IFN-γ, pg/ml) into culture medium (enzyme-linked immunosorbent assay (ELISA), BioLegend).

1. **Peptide apitope validation: presentation of allergen by fixed APC**

Splenocytes from HLA-DR4 mice were fixed with 0.5% paraformaldehyde in PBS for 5 min at 20^o^C, quenched with 0.4M glycine in PBS for 5 min and washed with cold PBS.

**Medium**: Resuspended cells were cultured in RPMI 1640 medium supplemented with 20mM HEPES, 100U/ml Penicillin, 100μg/ml streptomycin, 0.05μM 2-mercaptoethanol and 5% foetal calf serum cultured in a 96 well plate.

**Cells**:

- Fixed (test) or unfixed (control) splenocytes at 2x10^5^ cells/well together with
- Der p 1-specific T cell hybridoma cells at 1x10^5^cells/well

**Antigens:** Der p 1 at 50μg/ml or peptides at 100μg/ml

**Assay readout**: T-cell hybridoma activation was determined by IL-2 secretion after 24 hours of incubation with splenocytes and antigen at 37^o^C/5%CO_2_ (ELISA, BioLegend).

1. **Peptide apitope validation: direct binding of peptide to MHC Class II on CD11c+ cells *in vivo***

Direct binding of a potential peptide tolerogen to MHC Class II on APC was tested *in vivo*. HLA-DR4 mice received either a single 100μg dose of peptide D121B (test) or PBS alone (control), injected s.c. in the scruff of the neck. After 2h, spleens were harvested and CD11c^+^ dendritic cells (DC) isolated (microbeads, Miltenyi Biotec)

**Medium**: Resuspended cells were cultured in RPMI 1640 medium supplemented with 20mM HEPES, 100U/ml Penicillin, 100μg/ml streptomycin, 0.05μM 2-mercaptoethanol and 5% foetal calf serum cultured in a 96 well plate.

**Cells**:

- CD11c+ DCs at 0.5x10^5^ CD11c^+^ cells/well together with
- Der p 1-specific T cell hybridoma cells at 1x10^5^cells/well

**Control cultures:** either Der p 1, peptide D or analogues D111B, D121B (100μg/ml) antigens

**Assay readout**: T-cell hybridoma activation was determined by IL-2 secretion after 48 hours of incubation with CD11c+ DCs, +/- antigen at 37^o^C/5%CO_2_ (ELISA, BioLegend)

**S2.E: Statistical analysis**

The sample size calculation (95% power, 5% significance) for this study was based on a previous study(Bateman et al., 2008) and exceeded the minimum suggested by Lancaster(Lancaster, 2015) (n=30).

To compare PBMC responses to allergens, subjects were divided into HDM-positive (HDM-allergic) and HDM-negative individuals (combined groups non-HDM allergic and healthy

controls) in a cross-sectional study design, devised in R(Team, 2023). The experiment was conducted on a convenience sample, to undertake exploratory analysis. The variables were proliferation (SI), exposure to allergen (either HDM, Der p 1, peptide A, B, C, D or E) and length of exposure (day of sampling). A logarithmic transformation of SI was performed to improve variance stability (SI _log10_). A two-level regression was undertaken of log (SI) regressed on exposure group (HDM-positive or HDM-negative) and day of experiment (4, 6, or 8), with observations clustered within subjects. Only main effects were considered in this exploratory work. Due to a low PBMC yield from peripheral blood from one HDM-negative subject, insufficient cells were available for sampling on 3 different days of culture; PBMC cultures were omitted for day 4. Missing measurements were not associated with exposure to allergen and so assumed to be missing at random and therefore, not a source of bias in the multilevel analysis. For the analysis, each antigen was considered separately. Satterthwaite's method was used for t-tests, with significance set at *p<0.05, **p<0.01, ***p<0.001

For mouse tolerance experiments, data was analysed with an unpaired Student’s t-test with Welch’s correction using GraphPad Prism 9.0. Significance levels were set at *p<0.05, **p<0.005, ***p<0.0005.

**Table S2. Statistical analysis of PBMC responses to Der p 1 peptide allergens in HDM-positive and HDM-negative subjects**

| PBMC responses to Der p 1 peptide allergens | | | | | | | |
| --- | --- | --- | --- | --- | --- | --- | --- |
| Antigen | **SI _log10_ HDM+ vs HDM- (day 4,6,8)**  **t tests (Satterthwaite)** | | **SI**  **mean/antigen/day** | | | |  |
|  | **t-value** | **p** | **day** | **HDM-positive Mean (SD)** | **HDM-negative**  **Mean (SD)** | **p** |  |
| Der p 1 | 1.427 | 0.1607 | 4  6  8 | 3.03 (3.43)  7.69 (13.06)  8.12 (13.31) | 3.56 (5.54)  2.80 (3.19)  2.99 (3.78) | 0.698  0.110  0.103 |  |
| Peptide A | 0.583 | 0.5627 | 4  6  8 | 1.59 (0.63)  3.65 (6.78)  2.19 (2.70) | 2.91 (5.31)  1.84 (1.39)  1.78 (1.62) | 0.225  0.248  0.554 |  |
| Peptide B | 1.994 | 0.0525 | 4  6  8 | 3.81 (4.28)  9.84 (11.54)  7.12 (6.38) | 3.28 (3.47)  3.60 (2.92)  4.31 (4.93) | 0.661  0.023*  0.112 |  |
| Peptide C | 0.401 | 0.6904 | 4  6  8 | 1.96 (1.28)  3.78 (7.01)  2.41 (3.16) | 2.58 (3.57)  1.98 (1.83)  2.72 (4.24) | 0.425  0.272  0.784 |  |
| Peptide D | 2.633 | 0.0117* | 4  6  8 | 8.28 (9.49)  15.08 (17.80)  24.12 (44.19) | 5.01 (7.72)  2.81 (1.69)  5.67 (6.80) | 0.227  0.004**  0.072 |  |
| Peptide E | 0.404 | 0.6881 | 4  6  8 | 1.76 (1.04)  3.44 (8.52)  2.12 (2.97) | 2.34 (3.25)  2.03 (3.39)  1.48 (1.06) | 0.406  0.489  0.365 |  |
| PPD | 0.981 | 0.3319 | 4  6  8 | 33.84 (67.51)  72.51 (112.98)  47.97 (97.83) | 27.63 (43.14)  42.11 (78.79)  29.36 (53.14) | 0.728  0.313  0.449 |  |

For Satterthwaite t-tests, significance was set at *p<0.05, **p<0.01, ***p<0.001

(A)

| B analogues | Peptide aa sequence | Length (aa) |
| --- | --- | --- |
| B | PRGIEYIQHNGVVQESYYRYVAREQSCRRP | 30mer |
| B1  B2  B3  B4  B5  B6 | PRGIEYIQHNGVVQE  IEYIQHNGVVQESYY  IQHNGVVQESYYRYV  NGVVQESYYRYVAR  VQESYYRYVAREQSC  ESYYRYVAREQSCRRP | 15mer  15mer  15mer  15mer  15mer  16mer |

**Figure S1:** Fine mapping of T cell epitopes within Peptide B

To define potential T cell epitopes residing within peptide B, analogues B1-B6 were synthesised (15-16mer peptides, overlapping by 3aa, (A)) and used to re-challenge splenocytes isolated from HLA-DR4 mice immunised with Der p 1 (A) or PBS in CFA (B). T cell responses induced by B analogues in the *in vitro* assay were compared with stimulation by peptide B, Der p 1 or PPD antigen controls, as measured by IFN-γ secretion (pg/ml) after 72h in culture.

**Figure S2.** Ex vivo binding of Der p 1 antigens to CD11c+ cells following pre-loading in vivo

HLA-DR4 mice received either a single dose (s.c) of peptide D121B (test, orange bars) or PBS (control, blue bars). Two hours post injection, splenocyte CD11c+ cells were isolated and co-cultured with antigen (peptide D, D111B, D121B or Der p 1 at 100μg/ml or 10μg/ml) in the presence of Der p 1 specific T cell hybridoma cells for 48h *in vitro*. The T cell hybridoma was activated to secrete IL-2 in all cultures, whether pre-loaded with Der p 1 antigens or PBS. The graph is representative of 2 experiments.

**Supplementary References**

Bateman, E.A., Ardern-Jones, M.R., and Ogg, G.S. (2008). Identification of an immunodominant region of Fel d 1 and characterization of constituent epitopes. *Clin Exp Allergy* 38(11)**,** 1760-1768. doi: 10.1111/j.1365-2222.2008.03098.x.

Canaday, D.H. (2013). Production of CD4(+) and CD8(+) T cell hybridomas. *Methods Mol Biol* 960**,** 297-307. doi: 10.1007/978-1-62703-218-6_22.

Lancaster, G.A. (2015). Pilot and feasibility studies come of age! *Pilot Feasibility Stud* 1(1)**,** 1. doi: 10.1186/2055-5784-1-1.

Mazza, G., Ponsford, M., Lowrey, P., Campbell, M.J., Zajicek, J., and Wraith, D.C. (2002). Diversity and dynamics of the T-cell response to MBP in DR2+ve individuals. *Clin Exp Immunol* 128(3)**,** 538-547.

Team, R.C. (2023). "R: A language and environment for statistical

computing.". (Vienna, Austria: R Foundation for Statistical Computing).
